# LyTONEPAL: long term outcome of neonatal hypoxic encephalopathy in the era of neuroprotective treatment with hypothermia: a French population-based cohort

**DOI:** 10.1186/s12887-018-1232-6

**Published:** 2018-08-01

**Authors:** Thierry Debillon, Nathalie Bednarek, Anne Ego, the LyTONEPAL Writing Group, Pierre-Yves Ancel, Olivier Baud, Marie-Laure Charkaluk, Clothilde Desrobert, Cyril Flamant, Pierre Gressens, Gilles Kayem, Stephane Marret, Juliana Patkail, Loic Senthiles, Elie Saliba

**Affiliations:** 1 Neonatology Department, University Hospital Grenoble Alpes, Grenoble, France; 2 TIMC-IMAG, Grenoble Institute of Engineering, CNRS, Grenoble Alpes University, Grenoble, France; 3 Neonatology Department, University Hospital Alix de Champagne, Reims, France; 4 CReSTIC, Champagne-Ardennes University, EA3804 Reims, France; 5 Public Health Department, University Hospital Grenoble Alpes, Grenoble, France

**Keywords:** Late preterm and term births, Cohort, Population-based study, Hypoxic ischemic encephalopathy, Therapeutic hypothermia

## Abstract

**Background:**

Hypoxic-ischemic encephalopathy (HIE) is a rare neonatal condition affecting about 1‰ births. Despite a significant improvement in the management of this condition in the last ten years, HIE remains associated with high rates of death and severe neurological disability. From September 2015 to March 2017, a French national cohort of HIE cases was conducted to estimate the extent of long-term moderate and severe neurodevelopmental disability at 3 years and its determinants.

**Methods:**

This prospective population-based cohort includes all moderate or severe cases of HIE, occurring in newborns delivered between 34 and 42 completed weeks of gestation and admitted to a neonatal intensive care unit. Detailed data on the pregnancy, delivery, and newborn until hospital discharge was collected from the medical records in maternity and neonatology units. All clinical examinations including biomarkers, EEG, and imaging were recorded. To ensure the completeness of HIE registration, a registry of non-included eligible neonates was organized, and the exhaustiveness of the cohort is currently checked using the national hospital discharge database. Follow-up is organized by the regional perinatal network, and 3 medical visits are planned at 18, 24 and 36 months. One additional project focused on early predictors, in particular early biomarkers, involves a quarter of the cohort.

**Discussion:**

This cohort study aims to improve and update our knowledge about the incidence, the prognosis and the etiology of HIE, and to assess medical care. Its final objective is to improve the definition of this condition and develop prevention and management strategies for high-risk infants.

**Trial registration:**

NCT02676063. Date of registration (Retrospectively Registered): February 8, 2016.

## Background

### Hypoxic ischemic encephalopathy: Definition, incidence and risk factors

Hypoxic-Ischemic Encephalopathy is defined as the clinical manifestation of impaired neonatal brain function following asphyxia due to an antenatal and/or perinatal adverse event. Various criteria (biological, clinical and sometimes electrophysiological) are used to define the severity of the neurological damage [1], many of them being included in Sarnats’ original classification [2].

The incidence of HIE is currently imprecise with numbers ranging from 1 to 8 per 1000 live births worldwide [3, 4], but is nearer to between 1 and 2 per 1000 full term births in population-based studies conducted in high income countries [5–7]. In a regional population-based study in France, the prevalence of moderate or severe HIE per 1000 live births was estimated to be 0.86 (95% CI 0.61 to 1.10) in 2000 [8].

The identification of pregnancies at risk of intrapartum fetal asphyxia is a longstanding issue [9–13] and antepartum and intrapartum factors have been identified [5, 14]. Maternal pyrexia, a persistent occipito-posterior position, an acute intrapartum event (hemorrhage, maternal convulsions, uterine rupture, snapped cord, and birth before arrival at an obstetric facility), and instrumental vaginal delivery or emergency caesarean section are all labor-related events associated with HIE.

### Management of HIE and short and long-term outcomes

A meta-estimate of the proportion of death, cerebral palsy or motor/cognitive impairment (more than 2 standard deviations below the norm) was 47% (95% CI 36 to 57) at 3 years and older [15]. Among other outcomes identified during early childhood, 10–12% will develop post-neonatal epilepsy [16–21], 9% hearing loss or deafness, and 26% impaired vision or blindness [21].

Therapeutic hypothermia (TH) has been shown to safely improve the developmental outcome of infants with HIE [22–24], and reduces mortality or major neurodevelopmental disability at 18 months of age with a risk ratio (RR) of 0.75 (95% CI 0.68 to 0.83) [25]. Shankaran et al. reported a rate of death or survival with an IQ below 70 at 6 to 7 years of 47 and 62% in the hypothermia versus control groups, respectively [26].

French guidelines on TH for HIE infants were published by the French Society of Neonatology in 2010 [27], but a multicenter study has shown that the implementation of these guidelines has been heterogeneous [28].

### Predicting mortality and neurodevelopmental outcomes

The early prediction of morbidity and mortality is of crucial importance to initiate adequate neuroprotective care, early rehabilitation programs or eventually to discuss palliative care following the first week of life. Different tools derived from Sarnats’ clinical score [2] or Amiel-Tison’s score [29] have been proposed to predict short term morbidity and mortality. The Thompson score, which tests 9 independent clinical items is now increasingly used, considering its relevant predictive values at 12 and 24 months [30, 31].

Classification based on conventional electroencephalography (cEEG) signs, which play a significant role in making a prognosis, have been developed for full-term infants [32, 33]. A simplified version of the cEEG technique, amplitude-integrated EEG (aEEG) is widely used in northern Europe in neonatology, and allows cerebral monitoring from birth to several days of life. It appears as the most promising test in a recent meta-analysis of the prognostic values of current tests for neurodevelopmental outcomes (12–18 months) following perinatal asphyxia [34], with relevant performance as early as the first 6 h of life [35, 36].

While conventional Magnetic Resonance Imaging (MRI) is useful, it often only reveals subtle changes in response to injury during the first 5 days following perinatal asphyxia [37–39]*.* In contrast, diffusion weighted MRI gives more conspicuous findings which are seen earlier [40, 41]*.* In the meta-analysis of van Laerhoven (1306 full-term neonates), diffusion weighted MRI in the first week performed best in terms of specificity (0.89, 95% CI 0.62–0.98), while T1/T2 – weighted MRI (in the first 2 weeks) was highly relevant with excellent sensitivity (0.98, 95% CI 0.80–1.00) [34].

Finally, several potential biomarkers have been evaluated in the context of HIE: IL-6, an inflammatory cytokine produced by T-cells and macrophages [42, 43], cardiac troponin I (cTnI) a marker of ischemic myocardial muscle insult [44, 45], acylcarnitins, markers of mitochondrial dysfunction [46], and ischemia-modified albumin [47]. In addition, a French team has proposed matrix metalloproteinases (MMP) and their inhibitors (TIMP) as potential markers of the extent of HIE damage in newborns and consequently the prognosis [48].

Outstanding issues about HIE relate mainly to the long term moderate and severe neurodevelopmental outcomes, the early identification of children at risk, and the analysis of recent changes in medical practice. To our knowledge there is no recent or ongoing population-based study in developed countries addressing these questions [24, 49, 50]. The Long term prognosis of neonatal hypoxic encephalopathy in the era of neuroprotective treatment with hypothermia (LyTONEPAL) project is a national prospective cohort study funded by the French Ministry of Health. It received ethics committee in approval 2014 and was launched in 2015.

### Objectives

The main objective is the occurrence of death, or severe or moderate neurodevelopmental disability at 3 years of age, in a large population-based cohort of children born at full-term, or late preterm, with moderate or severe HIE. The study aims also to 1) identify the very early prognostic factors, including specific new biomarkers, of poor outcome at 3 years of age; 2) analyze the predictive value of the clinical examinations during the first weeks of life; 3) describe and evaluate the different neuroprotective treatment strategies, including TH.

The study of potential new biomarkers is the object of an ancillary study requiring early, non-routine, laboratory analyses: interleukin-6, metalloproteinase-9, TIMP-1, ischemia-modified albumin (a marker of hypoxia), troponin I, acylcarnitins and amino acids; assayed in the first 6 h of life and again at 72 h.

## Methods

### Study design

All French metropolitan regions and overseas administrative areas were invited to participate in the study, and a neonatologist from one level III neonatal intensive care unit (NICU) per region was appointed as the regional coordinating investigator. These physicians are almost always the heads of the neonatology departments of the regional university hospital. Senior professionals familiar with the long-term support of families of children with HIE in their respective centers are in charge of each center. A clinical research assistant is assigned to each region to collect data, check the exhaustive registration of HIE cases, and plan patient follow up.

Sixty-eight level III intensive care units in 22 French regions (22 of the 23 metropolitan regions and 2 overseas administrative areas) are participating in the study. One region representing 27,300 births (3.4%) of the 800,000 births registered each year in France refused to participate. Babies were included from September 2015 to March 2017. Nineteen centres participate to the ancillary study, and a quarter of the total cohort is involved in this project. Consecutive inborn and outborn neonates transferred to the NICU, were screened by the neonatologists who provided information to parents, registered informed consent and included the neonate in the cohort. As permitted by French regulations on clinical research, a datasheet of minimal information was completed in cases of refusal to participate or circumstances in which information delivery was difficult for ethical reasons (early neonatal deaths) or when the parents did not speak French. Participating centers were invited to adhere to current guidelines and perform all clinical examinations necessary for the optimal management of HIE. At discharge from the neonatology unit, the parents were informed of and asked to consent to a follow-up plan, to the age of 3 years, drawn-up on a regional basis.

### Participants

Eligible neonates were babies born at 34 weeks gestational age or more, presenting: 1) early neurological distress with clinical signs of moderate to severe HIE on standardized neurological examination performed by a senior investigator, 2) biological criteria of asphyxia during the first hour of life, including 2a) severe biological signs of asphyxia or 2b) moderate or no biological signs of asphyxia with perinatal adverse events (Table 1). Neonates with congenital malformations, chromosomal disorders and congenital neuromuscular disorders were not-included or subsequently excluded, as the pathogenesis of HIE requires several days to be confirmed.

**Table 1:** HIE neurological and biological inclusion criteria for the LyTONEPAL cohort

Neurological signs	• Moderate HIE: lethargy, hyper-reflexia, myosis, bradycardia, seizures, hypotonia with weak suck and poor Moro reflex **•** Severe HIE: stupor, flaccidity, small to mid-position pupils that react poorly to light, decreased stretch reflexes, hypothermia or absent Moro reflex
Biological criteria indicating asphyxia during the first hour after birth in a sample of umbilical-cord blood or any other blood sampled	• Severe biological signs: pH ≤7.0 or less or a base deficit ≥16 mmol per liter • Moderate/absent biological signs with additional perinatal events: ○ 7.0 < pH ≤ 7.15, or 10 ≤ a base deficit < 16 mmol per liter, or blood gas measurement unavailable ○ With: - an acute perinatal event (e.g. late or variable decelerations, cord prolapse, cord rupture, uterine rupture, maternal trauma, hemorrhage, or cardiorespiratory arrest) - or an abrupt change in fetal heart rate (FHR), defined as a persistent abnormal FHR after a period of normal tracing: bradycardia or prolonged deceleration, persistent variable decelerations, persistent late decelerations, and reduced heart variability - or either a 10-min Apgar score of 5 or less or assisted ventilation initiated at birth and continued for at least 10 min.

### Baseline data collection and follow-up organization

Neonatal data collection was done by neonatologists and clinical research assistants from the medical records of the mother and the neonate. Data included characteristics of the mother, her pregnancy and the circumstances of delivery, the neonate’s birth admission to the NICU, hospital stay in neonatal units, hospital discharge, and the organization of care (Table 2).

**Table 2:** Maternal and neonatal data collection until neonatal hospital discharge

Mother and pregnancy characteristics	• Maternal age, parity, educational level, occupation, history of previous pregnancies, medical history and complications of the current pregnancy
Circumstances of birth	• Labor and delivery mode: onset of labor, mode of delivery, intrapartum complications, fetal heart rate monitoring • Neonatal characteristics: gestational age, birthweight, gender, small for gestational age (<10th percentile), 1, 5 and 10 min Apgar scores, cord or arterial pH, base deficit within the first hour of life, neonatal transfer • Care in delivery room: O2, ventilation, resuscitation • Placental examination and fetal autopsy (if performed)
Admission in NICU	• Standardized neurological examination: Sarnat classification and Thompson score during the first week of life. • First clinical investigations: Electrophysiological examination (cEEG or aEEG) and neuro-imaging
Hospital stay in neonatal units	• Clinical investigations Electrophysiological examination (standard or amplitude-integrated), Cerebral ultrasound exams (Doppler and morphological), Brain MRI (diffusion –weighted and standard), Standard blood tests (heart, liver, kidney). • Treatment: analgesia, sedatives, anticonvulsant treatment, cooling or other neuroprotective strategies, ventilation, nutrition • Neonatal morbidity and mortality: pulmonary, cardiac, renal or liver pathologies, multi-organ system failure, neonatal death (date and presumed cause)
Hospital discharge from neonatal unit	• Standardized neurological examination: Sarnat definition, Thompson score, and Amiel-Tison neurological assessment. • Hospital discharge: discharge home, discharge to another care facility, death • Presumed circumstances or cause of HIE according to the neonatologist: Identified maternal, obstetrical, or neonatal conditions and events
Organization of care	• Place of birth, level of care of the maternity ward, timing of transfer and admission to NICU, transport conditions, level of care of the first and subsequent neonatal units, length of hospital stay

The follow-up plan was presented to families before hospital discharge. Follow-up includes three medical visits at 18, 24 and 36 months. At 6 month the mother is asked to fill in a questionnaire concerning post traumatic maternal stress [43]. At each visit information is collected on post-neonatal care (hospital admissions, medical visits, etc.), growth according to standardized anthropometric measurements, motor development, neurologic complications including seizures and all medical treatments. Motor disorders, especially cerebral palsy, are detected using the diagnostic criteria of the Surveillance of Cerebral Palsy in Europe (SCPE) network [44], based on the Gross Motor Function Classification System (GMFCS) [45]. The 18-month visit includes screening for autism [46] and a parental questionnaire concerning the home environment of the child and the family’s socio-economic status. Neurodevelopmental outcomes are assessed at 24 months using the revised Brunet Lezine test [47], the Age and Stages Questionnaire (ASQ) score [48], and a questionnaire screening for language disorders (“Inventaires Français du Développement Communicatif” adapted from the MacArthur communicative development inventories by Fenson et al.) [49]. The ASQ score is measured again at 36 months, and any sensorial impairment sought by an audiogram and an ophthalmological examination.

### Data management and completeness of HIE registration

Data are registered in an electronic case report form, with a secure interface. Parents are contacted by email at 6 months and requested to log-in to the same database to complete self-administered questionnaires. Data entry and quality control of responses and missing data are performed continuously.

Using the national hospital discharge database, we have defined an algorithm to seek unreported cases of HIE likely to have been eligible for inclusion in the cohort. This algorithm combines gestational age (≥34 weeks), date of hospital discharge, the diagnosis code for HIE, P91.6 in the International Classification of Disease 10th edition (recorded as the main or a related diagnosis), and hospital stay in a level III neonatology units of the participating regions. It excludes infants with chromosomal abnormalities (diagnosis code Q9*). This discharge database is crossed with the cohort database. For each newly suspected case admitted to a NICU, the investigating neonatologist is asked to consult the medical notes and check the inclusion criteria. If HIE is confirmed, these neonates are registered in the inventory of non-included eligible neonates with the collection of minimal data. These investigations are still in progress.

### Study outcomes

The primary outcome measure is a combined criterion, including death or moderate or severe neurodevelopmental deficiency at 3 years of age.

Severe neurodevelopmental deficiency is defined as:

- intellectual impairment (mental score > 2sd below the mean or intellectual quotient (IQ) < 70 according to the revised Brunet Lezine Score and ASQ scores),
- or cerebral palsy (GMFCS of 3–5),
- or sensorial impairment (bilateral blindness with vision < 20/200 acuity or deafness requiring amplification > 60 dB),
- or persistent disorder defined as recurrent seizures after discharge from the NICU requiring anti-convulsive therapy.

Moderate disability was defined as:

- intellectual impairment (1 sd < mental score ≤ 2 sd below the mean or 70 ≤ IQ < 85),
- cerebral palsy (GMFCS of 1–2),
- or hearing impairment requiring no amplification.

Secondary outcomes are the estimation of the relevance of very early prognostic factors, including the specific new biomarkers, clinical, biological, EEG and neuroimaging examinations during the first weeks of life, and the analysis of the effect of neuroprotective strategies, including therapeutic hypothermia.

### Sample size calculation

The sample size was calculated so as to demonstrate differences in prognosis according to risk factors. We expected a proportion of poor outcomes at 3 years of 70% versus 85% among unexposed versus exposed babies (difference rate of 15%, RR of 1.2). With 15% of newborns exposed to risk factors, the total sample size needed was 552 children followed-up at 3 years (with an alpha error of 0.05 and power of 0.80). We considered that the incidence of moderate or severe HIE might be around 1‰ births, corresponding to 800 cases of moderate or severe HIE per year in France, with a death rate of 20% before hospital discharge, a participation rate of 80% among survivors, and 10% patients lost to follow-up at 3 years. Consequently, the sample size of 552 estimated above was increased by a factor of 1.28, leading to a sample of 706 neonates with HIE. A 12 to 18 month period of recruitment was planned in order to reach this sample size.

### Statistical analysis

Children lost to follow-up will be compared to those enrolled with complete follow up for baseline characteristics. Subsequent analyses will be performed for each phase of the study, baseline data collection and follow-up.

Descriptive data summaries will be generated using means and standard deviations (or medians and inter-quartile range) for quantitative variables, and frequency distributions (total number, frequency and 95% CI) for qualitative variables.

Bivariate analyses will be performed to study the association between risk factors and short-term and long-term outcomes using analysis of variance (or a Kruskall-Wallis test) for continuous variables or Chi-square (or Fisher exact tests) for qualitative variables.

Adjusted relative risks and their 95% CI for the different outcomes will be determined using multilevel logistic regression analysis. A hierarchical logistic regression model will enable us to distinguish the individual (maternal and neonatal) variables from other variables associated with the organization of care such as the level of care in the maternity wards or the characteristics of the NICU, and to estimate the amount of variability within these levels.

The predictive ability of neonatal characteristics will be explored by estimating sensitivity, specificity, predictive values and likelihood ratios with their confidence intervals. The predictive accuracy concerning adverse outcomes will be defined as the proportion of correctly classified cases (sum of true positives and true negatives). The biological cut-off for quantitative biomarkers will be determined using Receiver Operating Characteristic (ROC) curves. Areas under the curve (AUC) of 90–100% will be considered as excellent and those between 80 and 90% considered as good. Analysis of simultaneous predictors of outcomes will be conducted using a stepwise process involving multivariable logistic regression and ROC curve analysis performed on the outcome probabilities obtained via logistic regression modeling.

*P*-values less than 0.05 will be considered statistically significant and statistical analysis will be conducted using Intercooled STATA (Version 13, Stata Corporation, College Station, TX, USA).

### Ethics approval and consent to participate

Because this study concerns newborn child, the information and the consent was obtained from both parents. For each eligible neonate, an information form was delivered to parents. A first oral consent to participate were requested by the neonatologist at the inclusion concerning the baseline data collection during hospitalization. If this consent was not obtained, the parents signed the information form to refuse their participation. In this case and according to French regulations, these neonates were registered in the inventory of non-included eligible neonates with the collection of anonymous minimal data.

At the end of the neonatal hospitalization a second but written consent was requested to participate to the standardized follow-up, and was signed by both parents and the neonatologist. Babies of parents who refused to participate were followed-up according to the usual practices of the center. According to French regulations, parents have a permanent right of access and rectification to their personal data, and can also leave the cohort at any time.

According the French law, the study protocol, including ethics and consent to participate, was approved by the Advisory National Committee on the treatment of personal health data for research purposes (Comité Consultatif sur le Traitement de l’Information en matière de Recherche sur la Santé, approval granted November 20, 2014; reference number 14.724. The authorizations were obtained:

- from the National French data protection authority (Commission Nationale Informatique et Libertés) on March 27, 2015; DR-2015-136
- and from the Regional Ethics committee CPP South-East V (Comité de Protection des Personnes Sud Est; Institutional Review Board n°5891) on July 18, 2014.

### Project governance

A national scientific and steering committee including pediatricians, obstetricians, perinatal epidemiologists and a project manager was constituted to organize the implementation and the general supervision of the cohort. At the regional level, a level III NICU hospital and a coordinating committee were appointed to handle the funding allotted to the region, and monitor recruitment and follow-up.

## Discussion

Late preterm and term newborns with HIE will face a range of medical, psychological, and social problems that raise specific questions at birth and thereafter. The need for information on the prevalence, causes, and consequences of HIE requires large population-based cohort studies with a long-term follow-up. Particularly because several changes in the management of HIE have occurred during the past decade and the risk factors for impaired neurodevelopment must be revisited.

Our cohort study offers an original approach because it will allow us to describe the initial clinical contexts linked to poor outcomes, including moderate disability. Moreover, in addition to full-term neonates, late preterm are included, a subgroup for which little data exists about HIE. The establishment and follow-up of this cohort and the collection of detailed data will increase our understanding about: 1) clinical, biological, electrophysiological and brain imaging predictors of long-term outcomes; 2) current care and management of HIE children in the light of recent progress in medical practices, and the particular role of the generalization of hypothermia treatment; 3) the organization of care and decision making in critical clinical situations.

Our findings should provide strong criteria to select the best candidates for neuroprotective strategies at the earliest possible stage. The increased knowledge we will gain about HIE prognosis may contribute to improving the relevance of information given to parents during the stay in the NICU, which is a crucial issue for neonatologists. Its importance is highlighted in the French regulations concerning pediatric intensive care. Moreover, it could provide a highly relevant contribution to ethical discussions and the current position regarding end of life decisions may need be updated in the light of the findings of our project.

## Abbreviations

aEEG: Amplitude-integrated electroencephalography
ASQ: Age and stages questionnaire
AUC: Area under the curve
cEEG: Conventional electroencephalography
CPP: Comité de protection des personnes
cTnl: Cardiac troponin I
GMFCS: Gross motor function classification system
HIE: Hypoxic-ischemic encephalopathy
IQ: Intellectual quotient
MMP: Matrix metalloproteinases
MRI: Magnetic resonance imaging
NICU: Neonatal intensive care unit
RR: Risk ratio
SCPE: Surveillance of cerebral palsy in Europe
TH: Therapeutic hypothermia
TIMP: Inhibitors of matrix metalloproteinases
